# Narbenhernien: minimalinvasive Operationsverfahren

**DOI:** 10.1007/s00104-023-02000-x

**Published:** 2023-12-09

**Authors:** Johannes Baur, Michael Meir

**Affiliations:** 1 Hernienzentrum Clarunis, Universitäres Bauchzentrum Basel, Standort St. Claraspital, Basel, Schweiz; 2 Klinik für Allgemein‑, Viszeral‑, Transplantations‑, Gefäß- und Kinderchirurgie, Universitätsklinkum Würzburg, Oberdürrbacherstr. 6, 97080 Würzburg, Deutschland

**Keywords:** Extended-total-extraperitoneale Technik, Mini- oder Less-open-Sublay-Technik, Endoskopisch assistierte Linea-alba-Rekonstruktion, Transabdominelle präperitoneale Patch-Technik, Intraperitoneales Onlay-Mesh, Extended totally extraperitoneal technique, Mini or less open sublay technique, Endoscopic-assisted linea alba reconstruction, Transabdominal preperitoneal patch technique, Intraperitoneal onlay mesh

## Abstract

**Hintergrund:**

In den letzten Jahren wurde eine Vielzahl neuer Operationstechniken zur minimalinvasiven Versorgung ventraler Hernien entwickelt und vorgestellt. In dieser Übersichtsarbeit werden diese minimalinvasiven Operationstechniken wie eTEP („extended totally extraperitoneal“), MILOS („mini or less open sublay“), ELAR (endoskopisch assistierte Linea-alba-Rekonstruktion), ventrale TAPP (transabdominelle präperitoneale Patch-Technik), IPOM (intraperitoneales Onlay-Mesh) Plus und LIRA (laparoskopische intrakorporale Rektusaponeuroplastie) vorgestellt und die hierzu relevanten bislang publizierten Ergebnisse präsentiert.

**Ergebnisse:**

Moderne minimalinvasive Techniken zur Behandlung ventraler Hernien bergen das Potenzial einer Reduktion von Wundinfektionen, geringerer postoperativer Schmerzen und einer kürzeren Krankenhausverweildauer im Vergleich zu den klassischen Hernienoperationen. Insbesondere Techniken mit retromuskulärer Netzposition sind aufgrund der Präparation in engen Räumen und aufgrund schwierig durchzuführender endoskopischer Nähte technisch anspruchsvoll und erfordern fundierte Kenntnisse der Anatomie der Bauchwand. Vor allem die Versorgung größerer Hernien sollte daher nur unter der Voraussetzung ausreichender Erfahrung und Fallzahl erfolgen.

**Schlussfolgerung:**

Die neuen, endoskopischen bzw. endoskopisch assistieren Verfahren zur Versorgung ventraler Hernien ermöglichen dem laparoskopisch erfahrenen Chirurgen, primäre und sekundäre ventrale Hernien minimal-invasiv zu versorgen.

## Abstract (en)

**Background:**

In recent years many new surgical techniques for minimally invasive treatment of ventral hernias have been developed and introduced. This review article presents these new minimally invasive surgical techniques, such as extended totally extraperitoneal (eTEP) repair, mini or less open sublay (MILOS), endoscopic-assisted linea alba reconstruction (ELAR), the ventral transabdominal preperitoneal patch (TAPP) technique, intraperitoneal onlay mesh (IPOM) plus and laparoscopic intracorporeal rectus aponeuroplasty (LIRA) and discusses recently published results.

**Results:**

Modern minimally invasive techniques for the treatment of ventral hernias have the potential to reduce surgical site infections, lower postoperative pain and lead to a shorter duration of hospital stay compared to the classical open hernia repair; however, especially techniques with a retromuscular mesh position are technically challenging due to the preparation in a limited space and difficult to perform endoscopic sutures and necessitate detailed knowledge of the anatomy of the abdominal wall. The treatment of larger hernias in particular should therefore only be carried out under the prerequisite of extensive experience and case numbers.

**Conclusion:**

The new endoscopic and endoscopically assisted techniques for treatment of ventral hernias enable the experienced laparoscopic surgeon to primarily and secondarily treat ventral hernias with minimally invasive techniques.

## Hintergrund

Die Rate an minimal-invasiven, endoskopischen Operationen hat in den letzten Jahren in Deutschland stetig zugenommen, und diese sind inzwischen auch bei onkologischen Operationen zunehmend als Standard etabliert. Ein großer Vorteil der minimal-invasiven Operationen ist, dass das Risiko einer postoperativen Narbenhernie deutlich reduziert wird. Das Risiko der Ausbildung einer Narbenhernie nach Medianlaparotomie wird in der Literatur mit 10–20 % angegeben [1, 2]. Zusätzlich führen minimal-invasive Operationsverfahren zu einer beschleunigten Rekonvaleszenz und einer Reduktion oberflächlicher Wundinfekte.

Mit zunehmender Expertise in endoskopischen Techniken wurde in den letzten Jahren eine Vielzahl minimal-invasiver Verfahren zur Behandlung ventraler Hernien entwickelt, um die Vorteile der minimal-invasiven Chirurgie auch für dieses Patientenkollektiv nutzen zu können.

Ziel der Techniken ist einerseits, eine möglichst anatomische Rekonstruktion der Bauchdecke zu erreichen, Spannung auf die Rekonstruktion der Bauchdecke zu reduzieren und andererseits das Zugangstrauma für den Patienten zu minimieren**.**

## Indikation

Die Indikation für eine minimal-invasive Versorgung von Narbenhernien ist nicht einheitlich definiert. Insgesamt ist die Entscheidung, welches Operationsverfahren bei welcher Hernie angewendet wird, sehr variabel. In einer dänischen Arbeit wurden 5 Hernienexperten über die von ihnen bevorzugte Verfahrenswahl bei 25 Patienten mit unterschiedlichen Hernienerkrankungen befragt. Es ergab sich dabei eine deutliche Divergenz in der Wahl des Zugangsweges (offen gegenüber laparoskopisch), der Wahl der Netzposition (Onlay, retromuskulär, intraperitoneal) und der Wahl der Netzfixation zwischen den 5 Chirurgen.

Die 2023 erschienene italienische Leitlinie zur Hernienreparation empfiehlt eine endoskopische Versorgung nur von Narbenhernien mit einem maximalen transversalen Durchmesser von 10 cm^2^. Allerdings ist die Datenlage dazu von niedriger Evidenz und die Empfehlung entsprechend schwach. Ähnlich äußert sich auch die Empfehlung der European Hernia Society (EHS; [3]).

Die meisten internationalen Leitlinien empfehlen aus diesem Grund bei der Wahl der Technik der Hernienreparation das Verfahren, mit dem der Chirurg am besten vertraut ist.

## Synopse der Operationsverfahren

Wie bei den offenen, klassischen Operationsverfahren kann auch bei den minimal-invasiven Verfahren zwischen unterschiedlichen Netzlokalisationen unterschieden werden ([4]; Tab. 1).

**Table 1:** 

Netzlokalisation	Akronym des Verfahrens
Onlay-/suprafasziale Verfahren	ELAR
Retromuskuläre/Sublay-Verfahren	MILOS
	eTEP, TES
Präperitoneale Verfahren	TAPP
Intraperitoneale Verfahren	IPOM
	IPOM Plus/LIRA

### ELAR – endoskopisch assistierte Linea-alba-Rekonstruktion

**Figure 1:**
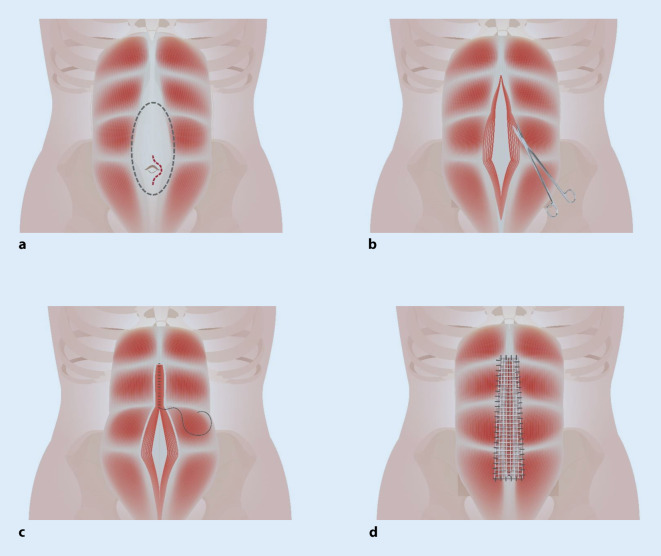

Das Verfahren wurde zur simultanen Versorgung einer umbilikalen Hernie mit simultaner Rektusdiastase oder mehreren epigastrischen Hernien im Bereich der Linea alba beschrieben [5]. Die Rektusscheide wird über einen Zugang im Bereich des Nabels nach kranial freipräpariert. Danach wird der mediale Anteil des vorderen Blattes der Rektusscheide beidseits ca. 1–2 cm lateral und ventral der Linea alba inzidiert. Eine neue Linea alba wird durch eine fortlaufende, nichtresorbierbare Naht der beiden medialen Ränder der durchtrennten Rektusscheiden rekonstruiert. Zur Verstärkung der Naht und als Ersatz für den Defekt im Bereich der vorderen Rektusscheide wird suprafaszial (Onlay-Position) ein Netz eingebracht und lateral fixiert (Abb. 1).

### eTEP/TES – „extendend totally extraperitoneal“/total endoskopisches Sublay

Bei der *eTEP(„extended total extraperitoneal“)-Technik* [6] erfolgt zunächst eine Inzision ca. 5 cm links lateral des Xiphoid auf Höhe des Rippenbogens. Das vordere Faszienblatt wird anschließend eröffnet und ein 12-mm-Trokar retromuskulär positioniert. Anschließend wird stumpf mit dem Endoskop der retromuskuläre Raum präpariert. Zwei weitere 5‑mm-Trokare werden ebenfalls im Bereich der lateralen linken Rektusscheide eingebracht. Dabei sind die Gefäß-Nerven-Bündel zu schonen. Es erfolgt sodann die vollständige Präparation des retromuskulären Raumes bis kaudal der Linea arcuata. Anschließend wird bei umbilikalen Hernien die mediale Begrenzung der linken Rektusscheide ca. 8 cm kranial des Nabels eröffnet (Crossover). Hierbei ist zu beachten, dass das Peritoneum dorsal der Linea alba intakt bleibt. Das präperitoneale Fett und das Peritoneum werden sodann von der Linea alba abpräpariert und so ein präperitonealer Raum kreiert. Ebenfalls supraumbilikal wird bei ausreichender Übersicht rechts lateral der Linea die hintere Rektusscheide inzidiert. Hierbei ist insbesondere bei ausgeprägter supraumbilikaler Rektusdiastase darauf zu achten, dass die Linea alba nicht irrtümlich inzidiert wird.

Nun wird der rechte retromuskuläre Raum präpariert. Somit sind ventral nun beide Rektusmuskeln sowie die Linea alba sichtbar. Dorsal befinden sich beide hinteren Rektusscheiden mit dem dazwischen befindlichen Peritoneum samt präperitonealem Fett. Die drei geschaffenen Räume (retromuskulärer Raum beidseits und präperitonealer Raum) werden in kraniokaudaler Richtung präpariert. Im weiteren Verlauf wird so der Hernienhals erreicht. Die Hernie wird sorgsam samt Bruchsack und präperitonealem Fett aus der Bruchlücke präpariert. Dabei ist die sehr empfindliche Nabelhaut insbesondere vor Diathermieschäden zu bewahren. Die Präparation setzt sich nun kaudal des Nabels fort, wo im weiteren Verlauf in das Cavum Retzii eingegangen wird. Nach hier erfolgter Präparation ist die Präparationsphase des Eingriffes abgeschlossen. Etwaige Läsionen der hinteren Schicht können anschließend mittels resorbierbarer Naht versorgt werden. Anschließend wird die Bruchlücke mittels knotenlosen Fadens verschlossen. Bei kleinen Brüchen erfolgt der Verschluss transversal, um das Risiko eines späteren Aufreißens der Bruchlücke zu verringern.

Die Naht der hinteren Rektusscheiden muss mit möglichst geringer Spannung angelegt werden

Bei epigastrischer Bruchlücke kann der Crossover auch kaudal des Nabels durchgeführt werden, um sich später von kaudal nach kranial der Bruchlücke zu nähern.

Bei Narbenhernien ist das Peritoneum häufig stark vernarbt. In diesen Fällen kommt es meist zu großen Defekten der hinteren Schicht. In diesem Fall können mittels fortlaufender, knotenloser Naht die beiden hinteren Rektusscheiden aneinandergenäht werden. Es ist jedoch darauf zu achten, dass diese Naht mit möglichst geringer Spannung durchgeführt wird. Bei zu hoher Spannung sind ein späteres Aufreißen der Naht und die Ausbildung einer intraparietalen Hernie möglich.

Ist die Naht nicht spannungsfrei möglich oder die Bruchlücke weist einen großen transversalen Durchmesser auf (≥ 8 cm), so ist die Durchführung eines Transversus-abdominis-Release (TAR) zu erwägen.

Nach Abschluss der Bauchdeckenrekonstruktion erfolgt die Einlage eines großporigen, nichtresorbierbaren Netzes. Dieses wird auf die hintere Schicht platziert und sollte die gesamte präparierte Fläche bedecken. Eine Fixierung des Netzes ist nicht erforderlich.

Auch bei der *total endoskopischen Sublay(TES)-Technik* erfolgt eine retromuskuläre Netzeinlage. Jedoch wird in diesem Falle zunächst eine Laparoskopie durchgeführt und die ipsilaterale Rektusscheide in deren lateralem Bereich inzidiert. Von dieser Inzision aus wird sodann der ipsilaterale retromuskuläre Raum präpariert, der Crossover samt Auslösung der Hernie durchgeführt und anschließend der kontralaterale retromuskuläre Raum dargestellt. Nach Verschluss der Bruchlücke und Einbringen des Netzes wird die initiale laterale Inzision der Rektusscheide wieder mittels knotenlosen Fadens verschlossen.

Nach Darstellung des hinteren Blattes der Rektusscheide wird der retromuskuläre Raum ähnlich der klassischen „total extraperitonealen Hernioplastik“ nach kranial und kaudal präpariert. Nach Präparation des Raumes werden die hinteren Blätter der Rektusscheide im Bereich des medialen Randes abgesetzt und das darunterliegende Peritoneum geschont. Der eigentliche Bruchsack wird abpräpariert und ggf. reseziert. Finden sich im Bruchsack ausgedehnte Verwachsungen, muss eine endoskopische Adhäsiolyse durchgeführt werden. Typischerweise wird dann der Defekt der Bauchwand ventral und dorsal mit mehreren knotenlosen Wundnähten endoskopisch verschlossen und damit die Bauchwand rekonstruiert. Abschließend wird ein großes Kunststoffnetz in den retromuskulären Raum eingebracht, der ggf. mit Kleber oder einzelnen Nähten fixiert wird.

**Figure 2:**
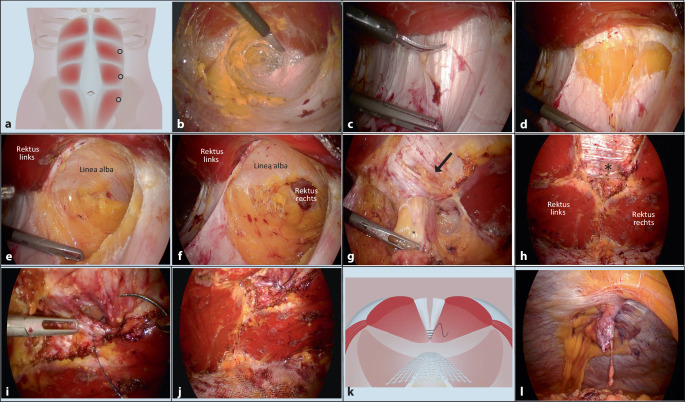

Die eTEP/TES ermöglicht damit die endoskopische Versorgung von Narbenhernien, epigastrischen Hernien und großen Nabelhernien (Abb. 2). Bei großen Defekten kann eine posteriore Komponentenseparation des Musculus transversus abdominis (Transversus-abdominis-Release, TAR) erfolgen, um die ventrale Bauchdecke mit einem großen Netz zu verstärken. Dies ist insbesondere dann notwendig, wenn der Verschluss der Bruchpforten endoskopisch nicht spannungsfrei möglich ist. Insbesondere bei Bruchlücken > 8 cm sollte eine TAR in Erwägung gezogen werden [7].

### MILOS – „mini/less open sublay“

Da sich die endoskopische Bruchsackpräparation bei der eTEP/TES manchmal komplex darstellt, können entweder primär oder sekundär eine offene Präparation des Bruchsackes und eine offene Adhäsiolyse erfolgen. Die Bezeichnung „mini“ bzw. „less open“ resultiert damit aus der reduzierten kutanen Wunde. Als Vorgabe der Entwickler des Verfahrens wird eine Inzision ≤ 5 cm als „mini“ sowie 6–12 cm als „less open“ bezeichnet [8].

Auch bei der MILOS-Operation ist eine posteriore Komponentenseparation möglich

Bei der klassischen MILOS-Operation wird durch den Bruchsack der Hernie primär offen operiert. Im Bereich des Defektes wird ein laparoskopischer Port eingebracht, der die retromuskuläre Implantation großer Netze ermöglicht. Nach klassischer Präparation des Bruchsackes und der Faszienränder wird der Bruchsack eröffnet. Intraabdominelle Adhäsionen können über die Hernie entweder offen oder laparoskopisch gelöst werden. Nach Resektion überschüssiger Bruchsackanteile wird das Peritoneum verschlossen, und es erfolgt die streng extraperitoneale Präparation der retromuskulären Schicht ähnlich der eTEP/TES. Auch bei der MILOS-Operation ist eine posteriore Komponentenseparation möglich.

### Ventrale TAPP – transabdominelle präperitoneale Patch-Technik

Zur minimal-invasiven Behandlung von Nabelhernien und epigastrischen Hernien wurde die transabdominelle präperitoneale Plastik (TAPP) der Leistenhernienchirurgie modifiziert. Zumeist erfolgt ein Zugang von lateral bzw. von suprasymphysär. Das Peritoneum wird wie für die inguinale TAPP eröffnet und ein ausreichender Raum zirkulär um die Hernie geschaffen. Entscheidend für die laparoskopische Präparation ist dabei ein zusätzlicher punktueller Druck von außen, um das Peritoneum mit laparoskopischen Instrumenten präparieren zu können. Nach Reposition des Bruchinhalts nach intraabdominell erfolgt ein laparoskopischer Verschluss der Bruchpforte mit einem knotenlosen, nichtresorbierbaren Faden. Ein nichtresorbierbares Netz wird präperitoneal platziert und fixiert (entweder mit Nähten oder mit resorbierbaren Ankern). Abschließend erfolgt ein lückenloser Verschluss des peritonealen Defektes mit einem resorbierbaren, knotenlosen Faden. Sollten weitere peritoneale Defekte im Rahmen der Präparation oder im Bereich der Bruchpforte entstanden sein, sollten diese ebenfalls verschlossen werden, um einen Kontakt des Netzes mit den Darmschlingen zu vermeiden.

Bei der TAPP bleibt die Integrität der myofaszialen Bauchdecke erhalten

Im Vergleich zu den Verfahren mit retromuskulärer Netzlage besteht der Vorteil bei dieser Technik darin, dass die Integrität der myofaszialen Bauchdecke erhalten bleibt und dennoch eine extraperitoneale Netzlage möglich ist. Jedoch ist der Erfolg dieser Methode sehr stark von den individuellen Gegebenheiten abhängig. Ist das Peritoneum sehr dünn oder sehr stark an der Bauchdecke adhärent, können auch sehr ausgeprägte Läsionen entstehen und das Peritoneum möglichweiser nicht mehr zur dorsalen Deckung eines Netzes dienlich sein.

### IPOM – intraperitoneales Onlay-Mesh

Die laparoskopische intraperitoneale Netzimplantation stellt aktuell weiterhin weltweit die häufigste endoskopische, minimal-invasive Technik zur Versorgung abdomineller Hernien dar. Allerdings ist in den letzten Jahren ein Rückgang dieser Methode in Deutschland zu verzeichnen [9]. Trotz Verbesserung der Netze mit Beschichtungen durch Kollagen ist die Entstehung prothetointestinaler Fistulierungen eine gefürchtete – wenn auch seltene – Komplikation dieser Hernienversorgung. Zusätzlich zeigt sich bei den Patienten durch traumatische Netzfixierung an der Bauchdecke eine erhöhte Rate akuter und chronischer Schmerzen [10]. Zudem sind beschichtete Netze und Einmalgeräte zur deren Fixierung (Tacker) ein erheblicher Kostenfaktor [11].

Typischerweise wird bei der klassischen IPOM-Versorgung ein beschichtetes Netz intraperitoneal aufgebracht und an der Bauchdecke durch transfasziale Nähte oder durch Spiraltacker in 2 konzentrischen Reihen (Double-crown-Technik) fixiert. Das Netz überbrückt damit im klassischen IPOM den Bruchdefekt, der nicht verschlossen wird.

### IPOM Plus und LIRA – laparoskopische intrakorporale Rektusaponeuroplastie

Verschiedene Arbeiten haben gezeigt, dass ein zusätzlicher Verschluss der Bruchpforte die Rate an Rezidiven, Serombildung und Pseudorezidiven deutlich reduzieren kann [12–14]. Aus diesem Grund wurden verschiedene Techniken etabliert, die eine Präparation des Bruchsackes und einen Verschluss des Defektes ermöglichen. Bei der IPOM-Plus-Technik wird dabei ein kleiner Defekt (< 3 cm) mit einem knotenlosen Faden verschlossen und anschließend das Netz intraperitoneal platziert und wie beim klassischen IPOM fixiert.

Bei größeren Defekten kann eine intrakorporale Rektusaponeuroplastie (LIRA) verwendet werden [15]. Das hintere Blatt der Rektusscheide wird beidseits lateral der Linea alba beidseits durchtrennt. Die dadurch entstandene Mobilität der hinteren Rektusscheide wird verwendet, um Defekte bis 10 cm mit einem knotenlosen Faden zu verschließen (ähnlich einem posterioren myofaszialen Release siehe ELAR). Nach Defektdeckung wird erneut das Netz intraperitoneal platziert und wie beim klassischen IPOM fixiert.

## Ergebnisse

Insgesamt gibt es in der Literatur in den letzten Jahren eine deutliche Zunahme der Publikationen, die die neuen endoskopischen Techniken zur Versorgung ventraler Hernien untersuchen. Bei den meisten Studien handelt es sich jedoch um retrospektive Beobachtungs- oder Vergleichsanalysen niedriger Evidenz. Die ersten randomisierten Studien zeigen beim Vergleich eTEP und IPOM jedoch eine signifikante Reduktion der postoperativen Schmerzen. Auch kann durch die Anwendung der eTEP trotz deutlich längerer Operationsdauer über die Hälfte an Kosten eingespart werden [11, 16]. Insbesondere für eine genaue Evaluation der Langzeitergebnisse ist die aktuelle Datenlage noch unzureichend.

Ältere Übersichtsarbeiten und Metaanalysen haben vor allem das klassische IPOM-Verfahren mit offenen Operationsverfahren verglichen und kamen zu dem Schluss, dass insbesondere die Reduktion von Wundinfekten für die minimal-invasive Operation spricht. Allerdings zeigten die Verfahren eine ähnliche Rate chronisch postoperativer Schmerzen [17, 18]. Eine aktuelle Metaanalyse hat aus diesem Grund die eTEP mit dem klassischen IPOM-Verfahren verglichen und postuliert, dass die eTEP-Patienten durch geringere postoperative Schmerzen sowie einen kürzeren Krankenhausaufenthalt profitieren [19]. Ähnliche Ergebnisse hat eine aktuelle Auswertung von Registerdaten über 5 Jahre von den Entwicklern des MILOS-Verfahrens im Vergleich zum klassischen IPOM und der offenen Narbenhernienreparation gezeigt. So zeigen die 5‑Jahres-Daten im Herniamed-Register für das MILOS-Verfahren eine signifikante Verbesserung postoperativer Schmerzen, eine Reduktion der postoperativen Komplikationen, eine geringere Rate an Rezidiven im Vergleich zu den klassischen Verfahren [10]. Ein weiterer potenzieller Vorteil des MILOS-Verfahrens im Vergleich zur eTEP könnte ein besseres kosmetisches Ergebnis sein, da das MILOS-Verfahren eine Reduktion überschüssiger Haut im direkten Bruchbereich erlaubt.

Die modifizierten IPOM-Verfahren IPOM Plus und LIRA wurden ebenfalls vor allem mit dem klassischen IPOM-Verfahren verglichen. In den ersten Studien wurde ein Vorteil von IPOM Plus gegenüber dem klassischen IPOM durch geringeres „Bulging“, geringere Serombildung und ein geringeres Rezidivrisiko postuliert [20, 21]. Dies konnte allerdings in einer aktuellen multizentrischen, prospektiven Studie nicht bestätigt werden [22]. Die spanischen Entwickler der LIRA-Technik postulieren jedoch in einer Kohortenstudie bessere Ergebnisse für den posterioren myofaszialen Release im Vergleich zur IPOM-Plus-Technik [23].

## Schlussbetrachtung

Zusammenfassend sind in den letzten Jahren einige neue Verfahren entwickelt worden, die eine minimalinvasive, endoskopische Hernienversorgung bei ventralen Hernien ermöglichen und nicht zu einer höheren Rate an Rezidiven der Hernien führen. Zu betonen ist, dass viele der Verfahren jedoch eine hohe endoskopische bzw. laparoskopische sowie anatomische Expertise des Operateurs voraussetzen. Die Präparation erfolgt zumeist in engem Raum, ggf. ist eine aufwendige laparoskopische Adhäsiolyse notwendig. Zusätzlich müssen Defekte endoskopisch genäht werden, was durch die eingeschränkte Mobilität klassischer laparoskopischer Instrumente erschwert wird.

Wichtig ist, dass der Operateur bei der Planung und Durchführung der Versorgung einen intraoperativen Verfahrenswechsel in Erwägung zieht und mit dem Patienten präoperativ bespricht. So kann bei schweren Verwachsungen im Bruchsack eine eTEP in ein MILOS-Verfahren gewechselt werden, da insbesondere Verwachsungen im Bereich des Bruchsackes manchmal laparoskopisch schwer zu lösen sind. Auch kann es bei Defekten im Bereich des Peritoneums und des hinteren Faszienblattes ggf. notwendig sein, ein beschichtetes Kunststoffnetz zu verwenden. Schließlich sollte die Konversion auf eine offene Hernienversorgung immer Bestandteil des Aufklärungsgespräches sein.

## Fazit für die Praxis

- Die aktuellen Techniken wie „extended totally extraperitoneal“ (eTEP), „mini or less open sublay“ (MILOS), „endoscopic-assisted linea alba reconstruction“ (ELAR), ventraler „transabdominal preperitoneal patch“ (TAPP), „intraperitoneal onlay mesh“ (IPOM) Plus und „laparoscopic intracorporeal rectus aponeuroplasty“ (LIRA) ermöglichen dem laparoskopisch erfahrenen Chirurgen, Hernien minimal-invasiv zu operieren.
- Diese minimal-invasiven Operationen sind für die Patienten mit einer Reduktion oberflächlicher Wundinfekte, einer Reduktion postoperativer Schmerzen und einem verkürzten Krankenhausaufenthalt verbunden.
- Durch die Einschränkungen der Beweglichkeit der laparoskopischen Instrumente, die Enge des präparatorischen Raumes ist die Versorgung auf nichtkomplexe Hernien (< 10 cm Durchmesser) beschränkt.
